# Prevalence and determinants of compassion fatigue among nurses in a district hospital in Bangladesh

**DOI:** 10.1186/s12912-026-04736-3

**Published:** 2026-05-08

**Authors:** Mansur Helal Sajid, Pothik Hossain, Ridwane Sharife, Farzana Haque

**Affiliations:** Department of Nursing and Health Science, Jashore University of Science and Technology, Jashore, Bangladesh

**Keywords:** Compassion fatigue, Nurses, Professional quality of life, Burnout, Organizational support, District hospital, Bangladesh

## Abstract

**Background:**

Compassion fatigue is an occupational hazard among nurses that arises from prolonged exposure to patient suffering and to work-related stress. In resource-limited healthcare settings, such as public district hospitals in Bangladesh, high patient loads, staffing shortages, and limited organizational support may increase nurses’ vulnerability to CF. However, evidence from district-level hospitals in low- and middle-income countries is limited.

**Methods:**

A cross-sectional study was conducted between January and June 2025 among nurses working at a 250-bedded district hospital in Bangladesh. Using stratified random sampling, 380 nurses from the medicine, surgery, pediatrics, and intensive care/emergency units were recruited. Compassion fatigue was assessed using the Professional Quality of Life Scale (ProQOL-5). Descriptive statistics were used to estimate the prevalence. Chi-square tests and multivariate logistic regression were performed to identify the demographic and occupational predictors of high compassion fatigue.

**Results:**

Overall, 44.2% of nurses reported high compassion fatigue, and 37.6% reported moderate levels. The highest prevalence was observed among nurses working in intensive care/emergency units (52%). Multivariate analysis identified lack of organizational support (odds ratio [OR] 2.6, 95% confidence interval [CI] 1.6–4.1), more than 10 years of service (OR 2.3, 95% CI 1.4–3.8), and intensive care/emergency assignment (OR 1.9, 95% CI 1.2–2.9) as significant predictors of high compassion fatigue scores. Subscale analyses indicated higher burnout and secondary traumatic stress among nurses working in high-acuity departments.

**Conclusions:**

Compassion fatigue is highly prevalent among nurses in a resource-limited district hospital in Bangladesh, particularly among those working in high-acuity settings and those reporting insufficient organizational support. These findings highlight the need for targeted organizational strategies, including improved support systems, workload management, and mental health interventions, to promote nurses’ well-being and sustain quality patient care in similar healthcare contexts.

## Introduction

Nurses are a fundamental component of healthcare systems and are central to delivering safe, effective, and compassionate care. In Bangladesh, this role is particularly pronounced in public district hospitals, which serve large catchment populations and frequently operate under resource constraints. Nurses working in these settings are often required to manage high patient loads, work for prolonged hours, and receive limited institutional support, all of which contribute to sustained occupational stress.

A growing body of evidence indicates that prolonged exposure to demanding care environments places nurses at risk for compassion fatigue. Compassion fatigue refers to emotional and physical exhaustion arising from continuous engagement with patients suffering from trauma or serious illness. It has been conceptualized as a form of secondary traumatic stress resulting from indirect exposure to trauma through caregiving relationships rather than direct personal experience [1]. Compassion fatigue is distinct from burnout; while burnout typically develops gradually as a response to chronic workplace stress, compassion fatigue may occur more abruptly and is closely linked to empathetic engagement with patients’ distress [2].

In line with the Professional Quality of Life Model, compassion fatigue is conceptualized as a multidimensional construct comprising burnout and secondary traumatic stress, balanced against compassion satisfaction. Burnout reflects cumulative occupational stress, whereas secondary traumatic stress arises from indirect exposure to patients’ trauma. This distinction clarifies the mechanisms through which workplace and organizational factors influence nurses’ professional quality of life [3].

International studies suggest that compassion fatigue affects a substantial proportion of the nursing workforce, with prevalence estimates ranging between 30% and 50% across different healthcare contexts [4–6]. This condition has been associated with a range of adverse outcomes, including emotional distress, diminished job satisfaction, reduced work engagement, increased absenteeism, and heightened intention to leave the profession [2–8]. Importantly, compassion fatigue may also have implications for patient care because emotional exhaustion and reduced empathic capacity can compromise communication, clinical judgment, and the overall quality of care.

Furthermore, evidence indicates that compassion fatigue is not evenly distributed across different clinical settings. Nurses working in high-acuity environments, such as intensive care units and emergency departments, consistently report higher levels of compassion fatigue than those working in lower-intensity settings [6, 9]. Frequent exposure to critically ill patients, life-threatening situations, and traumatic events contributes to the cumulative emotional burden in these departments.

In Bangladesh, the risk of compassion fatigue among nurses may be amplified by the broader challenges facing the health system. Public hospitals commonly experience staffing shortages, overcrowded wards, and limited access to psychosocial support services (SPSs). Recent studies conducted in the Bangladeshi context have documented elevated levels of burnout, sleep disturbances, and psychological distress among healthcare workers, particularly during the COVID-19 pandemic [10–12]. These findings highlight the vulnerability of the nursing workforce and underscore the need for future studies to examine compassion fatigue as a distinct but related occupational phenomenon.

Despite its relevance, empirical research on compassion fatigue among Bangladeshi nurses remains limited. Existing studies have predominantly focused on burnout or general mental health outcomes and have often been conducted in tertiary care or pandemic-specific settings [10–12]. However, most of these studies are concentrated in specialized or urban healthcare facilities, with limited attention to district-level hospitals, which serve as primary access points for a large proportion of the population and operate under significant resource constraints. These settings differ substantially in terms of staffing patterns, workload intensity, and availability of organizational support, which may uniquely influence nurses’ vulnerability to compassion fatigue. Moreover, the organizational and occupational determinants of compassion fatigue in such resource-limited environments have not been adequately explored.

Against this background, the present study aimed to assess the prevalence and determinants of compassion fatigue among nurses working in a district hospital in Bangladesh. By examining demographic, occupational, and organizational factors, this study provides context-specific evidence to inform nursing management practices, organizational policy development, and targeted interventions to promote nurses’ well-being and sustain quality patient care in resource-constrained healthcare settings.

## Background and literature review

Compassion fatigue has been increasingly recognized as a significant occupational phenomenon affecting healthcare professionals routinely exposed to patient suffering, trauma, and emotional distress. The concept was first articulated by Figley, who described compassion fatigue as a form of secondary traumatic stress experienced by caregivers through indirect exposure to others’ trauma [1]. Unlike general occupational stress, compassion fatigue is closely linked to the empathic engagement required in caregiving roles and reflects the emotional cost of caring for individuals who experience pain, illness, or psychological trauma.

Within the nursing profession, compassion fatigue has been conceptualized as a multidimensional construct encompassing elements of burnout, secondary traumatic stress, and compassion satisfaction, as operationalized by the Professional Quality of Life (ProQOL) model [3]. Burnout reflects emotional exhaustion, frustration, and reduced professional efficacy, whereas secondary traumatic stress refers to stress symptoms resulting from indirect exposure to traumatic experiences in the workplace. In contrast, compassion satisfaction represents the positive aspects of caregiving, including the fulfillment and meaning derived from helping others. An imbalance among these components is believed to contribute to the development and severity of CF.

A growing body of international literature documents the prevalence of compassion fatigue among nurses. Systematic reviews and meta-analyses indicate that between 30% and 50% of nurses report moderate to high levels of compassion fatigue, with variability across clinical settings and healthcare systems [4, 5]. Studies conducted in high-acuity environments consistently demonstrate higher prevalence rates, particularly among nurses working in intensive care units, emergency departments, oncology units, and palliative care settings [6, 9]. These findings suggest that repeated exposure to critically ill patients, life-threatening situations, and emotionally charged clinical encounters play a central role in the development of CF.

Research has identified a range of individual and occupational factors associated with CF. Longer duration of professional service, extended working hours, high patient-to-nurse ratios, and inadequate organizational support have been repeatedly linked to an increased risk of developing compassion fatigue [2, 7]. Organizational factors, including leadership support, availability of mental health resources, and workplace culture, have emerged as particularly important determinants, highlighting that compassion fatigue is not solely an individual-level issue but is strongly influenced by the broader work environment [7, 14].

In low- and middle-income countries, healthcare system constraints may further intensify nurses’ vulnerability to compassion fatigue. Resource limitations, staffing shortages, and high patient volumes are common challenges in public healthcare facilities that may contribute to the sustained occupational stress. Evidence from South Asia and other developing regions indicates that nurses working in resource-constrained settings often experience higher levels of burnout and psychological distress than their counterparts in high-income countries [13]. However, compassion fatigue, as a distinct construct, has received comparatively less empirical attention in these contexts.

In Bangladesh, existing research has primarily focused on burnout, sleep disturbances, and general mental health outcomes among healthcare workers, particularly during the COVID-19 pandemic [10–12]. These studies have highlighted the substantial psychological burden on nurses and other healthcare professionals, underscoring the vulnerability of the workforce. Nevertheless, few studies have specifically examined compassion fatigue among nurses, and those that do exist are largely limited to tertiary care or pandemic-related settings. Evidence from district-level hospitals, which are the backbone of public healthcare delivery in Bangladesh, is scarce [15, 16].

Furthermore, most available studies in the Bangladeshi context have emphasized individual-level factors, with limited exploration of the organizational and departmental determinants of CF. This represents a critical gap, as organizational conditions play a central role in shaping nurses’ professional quality of life. Addressing this gap is essential for developing effective, context-sensitive interventions that move beyond individual coping strategies to system-level solutions.

In light of these limitations, this study seeks to contribute to the existing literature by examining compassion fatigue among nurses working in a district hospital in Bangladesh. By integrating demographic, occupational, and organizational factors within a theory-informed framework, this study aims to provide a more comprehensive understanding of compassion fatigue in a resource-limited healthcare setting and generate evidence that can inform nursing management, policy development, and future intervention strategies.

Despite generally consistent findings, prior studies show variability in the relative importance of organizational versus individual determinants, and limited evidence from resource-constrained district hospital settings suggests that these relationships may differ across contexts. This highlights the need for context-specific investigation.

### Conceptual framework

**Fig. 1:**
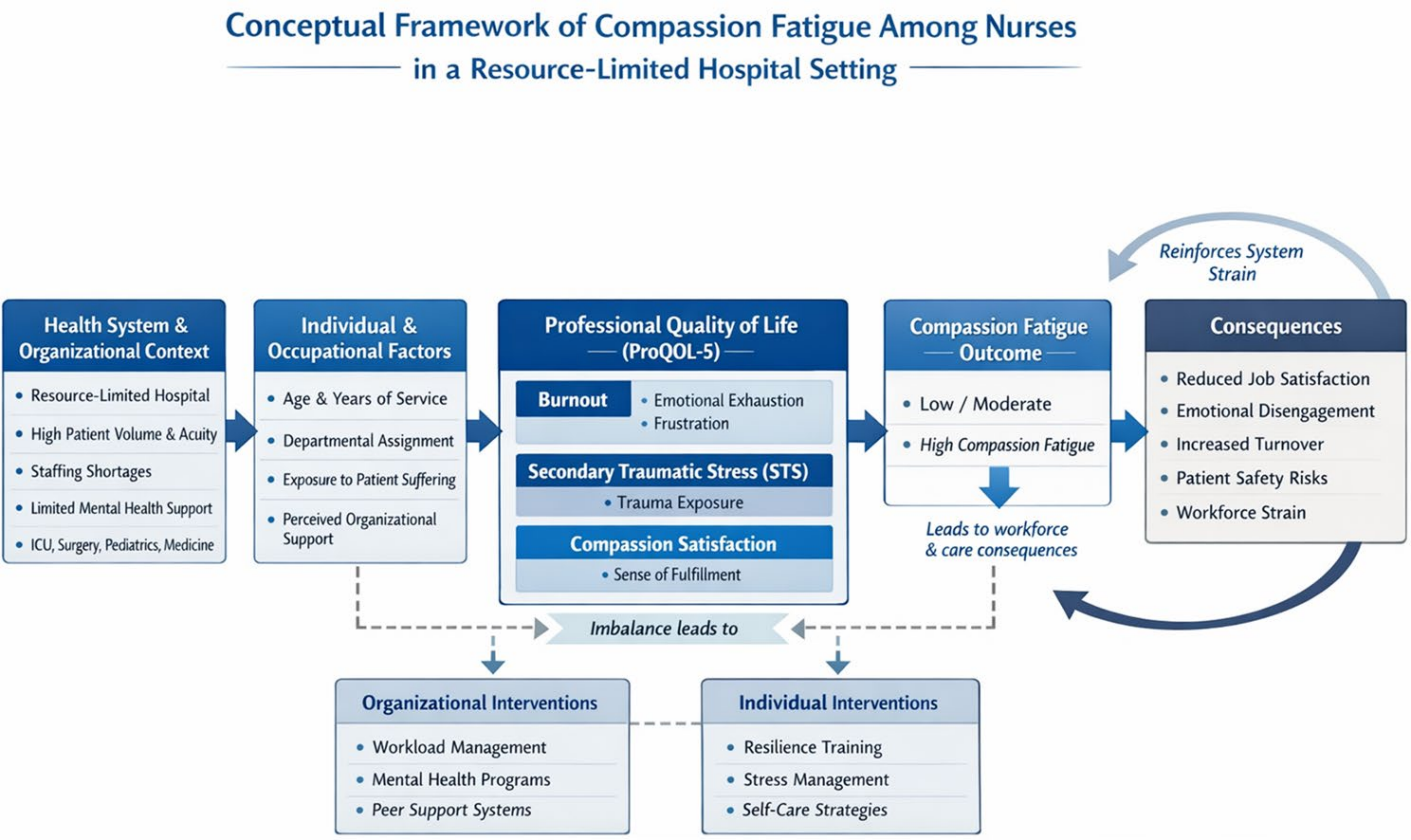
Conceptual framework of compassion fatigue among nurses

This study was guided by a conceptual framework informed by Figley’s compassion fatigue theory and the Professional Quality of Life (ProQOL) model. The framework conceptualizes compassion fatigue as a multidimensional outcome resulting from the interaction of organizational context, occupational exposure, and individual characteristics within resource-limited healthcare settings. Health system and organizational factors, such as staffing constraints, departmental workload intensity, and perceived organizational support, interact with individual characteristics, including age, years of service, and departmental assignment. These influences are mediated through burnout, secondary traumatic stress, and compassion satisfaction, leading to varying levels of compassion fatigue and associated workforce and patient care consequences (Fig. 1).

In this framework, demographic and organizational factors are conceptualized as independent variables influencing compassion fatigue through mediating components of burnout, secondary traumatic stress, and compassion satisfaction.

These mediating components were included based on the Professional Quality of Life Model, which identifies burnout, secondary traumatic stress, and compassion satisfaction as the core dimensions through which occupational and organizational factors influence compassion fatigue.

## Methods

### Study design and setting

A cross-sectional study design was employed to assess the prevalence and determinants of compassion fatigue among nurses. The study was conducted at the Jashore 250-Bedded General Hospital, a public district-level hospital located in the Khulna Division of Bangladesh. This hospital serves as a major referral center for the surrounding districts and provides a wide range of medical and surgical services to the patients. Nurses working in this setting routinely manage high patient volumes under resource-constrained conditions, making it an appropriate context for examining compassion fatigue.

Data collection was conducted between January and June 2025. A cross-sectional approach was selected as it is commonly used in occupational health and nursing workforce research to estimate prevalence and explore associations between outcomes and potential risk factors within a defined population at a specific point in time [13].

### Sample size calculation

The required sample size was calculated using the standard formula for estimating a single-population proportion. A prevalence (p) of 45% was assumed based on the findings of previous studies and a pilot assessment [4]. A 95% confidence level (Z = 1.96) and margin of error (d) of 0.05 were applied. The minimum calculated sample size was 381 participants in total.

To account for potential non-response, an additional 10% was added, resulting in a target sample of 424. Of these, 380 nurses completed the survey, yielding an adequate sample size for the statistical analysis.

### Pilot study

Prior to the main data collection, a pilot study was conducted with 30 nurses at the study hospital. The pilot study aimed to assess the clarity, comprehensibility, and internal consistency of the questionnaire. Minor wording adjustments were made based on the participants’ feedback to improve clarity. The reliability of the Professional Quality of Life Scale (ProQOL-5) demonstrated acceptable internal consistency, with a Cronbach’s alpha coefficient of 0.78.

Data from the pilot study were excluded from the final analysis.

### Sampling procedure and eligibility criteria

Stratified random sampling was used to ensure representation of key clinical departments. Nurses were stratified according to their departmental assignments: Medicine, Surgery, Pediatrics, and Intensive Care/Emergency. Participants were randomly selected from each stratum proportionate to the departmental size. Nurses were eligible for inclusion if they were registered nurses employed at the hospital, had at least one year of clinical experience, and provided informed consent to participate.

### Data collection instrument

Data were collected using a structured, self-administered questionnaire consisting of two main components: The first component gathered demographic and occupational information, including age, sex, years of service, and department of assignment. The second component comprised the Professional Quality of Life Scale (ProQOL-5), a widely used and validated instrument designed to assess compassion fatigue and related constructs.

The ProQOL-5 includes subscales measuring burnout, secondary traumatic stress, and compassion satisfaction. Higher burnout and secondary traumatic stress scores indicate greater emotional strain, whereas higher compassion satisfaction scores reflect greater professional fulfillment. A ProQOL-5 score of 30 or higher was used to classify high compassion fatigue.

Compassion fatigue was measured using the **Professional Quality of Life Scale (ProQOL-5)**, a previously published and widely validated instrument designed to assess burnout, secondary traumatic stress, and compassion satisfaction among helping professionals in the field. The ProQOL-5 has been used extensively in nursing and healthcare research across diverse settings and populations [3].

The cut-off score (≥ 30) was determined in accordance with the Professional Quality of Life Scale (ProQOL-5) guidelines and supported by prior studies employing similar classification thresholds [2, 3].

### Data collection procedure and ethical considerations

Data were collected by trained research assistants. Participants were informed about the purpose of the study, the voluntary nature of participation, and their right to withdraw at any time without any consequences. Written informed consent was obtained from all participants before data collection. Confidentiality and anonymity were strictly maintained throughout the study period.

Ethical approval was obtained from the Institutional Review Boards of the Jashore University of Science and Technology and Jashore 250-Bedded General Hospital (Approval No. JGH/2025/045). All procedures were performed in accordance with the Declaration of Helsinki.

### Data analysis

Data were entered and analyzed using SPSS (version 26). In addition to statistical analyses, data visualization was performed using **RStudio** (version 2026.01.0-392; R version 4.5.2). Graphical representations were generated to enhance the clarity and interpretability of the results, ensuring consistency with the analyzed data. Descriptive statistics were used to summarize the demographic characteristics and levels of compassion fatigue. Means and standard deviations were calculated for continuous variables, and frequencies and percentages were used for categorical variables.

Associations between compassion fatigue and demographic or occupational factors were initially examined using chi-square tests. Variables with statistical significance were entered into a multivariate logistic regression model to identify the independent predictors of high compassion fatigue. Results are reported as odds ratios (ORs) with corresponding 95% confidence intervals (CIs). Statistical significance was set at *P* < 0.05.

Multicollinearity among independent variables was assessed using Variance Inflation Factor (VIF), with values below 5 considered acceptable. Model fitness was evaluated using the Hosmer–Lemeshow goodness-of-fit test [17].

Variables with *p* < 0.05 in bivariate analysis were included in the multivariate logistic regression model.

## Results

### Demographic characteristics

A total of 380 nurses participated in this study. The majority of the participants were female (91%), while male nurses constituted 9% of the sample. The mean age of the participants was 29.4 ± 5.8 years. The mean duration of professional service was 7.2 ± 4.1 years.

**Fig. 2:**
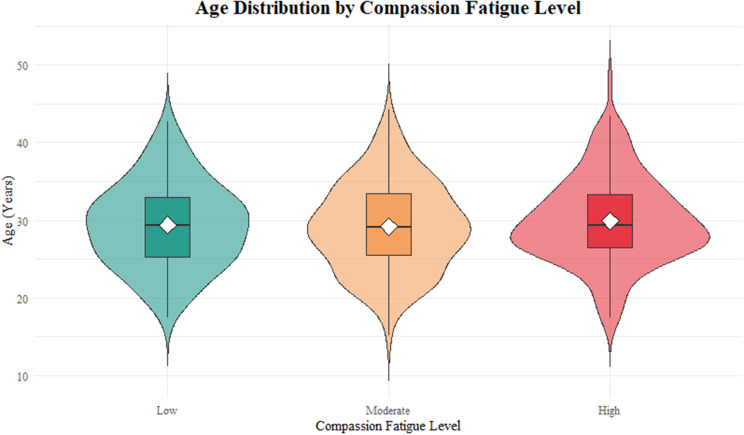
Age distribution by compassion fatigue level

Participants were distributed across four clinical departments: Medicine (28%), Surgery (25%), Pediatrics (22%), and Intensive Care/Emergency (25%). The age distribution by compassion fatigue level is illustrated in Fig. 2, which demonstrates a shift toward older age groups among nurses reporting high compassion fatigue.

### Prevalence of compassion fatigue

High compassion fatigue was reported by 44.2% of the nurses (*n* = 168). Moderate compassion fatigue was observed in 37.6% (*n* = 143), while 18.2% (*n* = 69) reported low levels. These findings indicate that more than four-fifths of the participants experienced at least moderate compassion fatigue.

Table 1 presents the demographic characteristics of the participants. Multivariate logistic regression identified significant predictors of high compassion fatigue.

**Table 1:** Prevalence of compassion fatigue among nurses (*n* = 380)

Level	Frequency	Percentage (%)
High	168	44.2
Moderate	143	37.6
Low	69	18.2

**Fig. 3:**
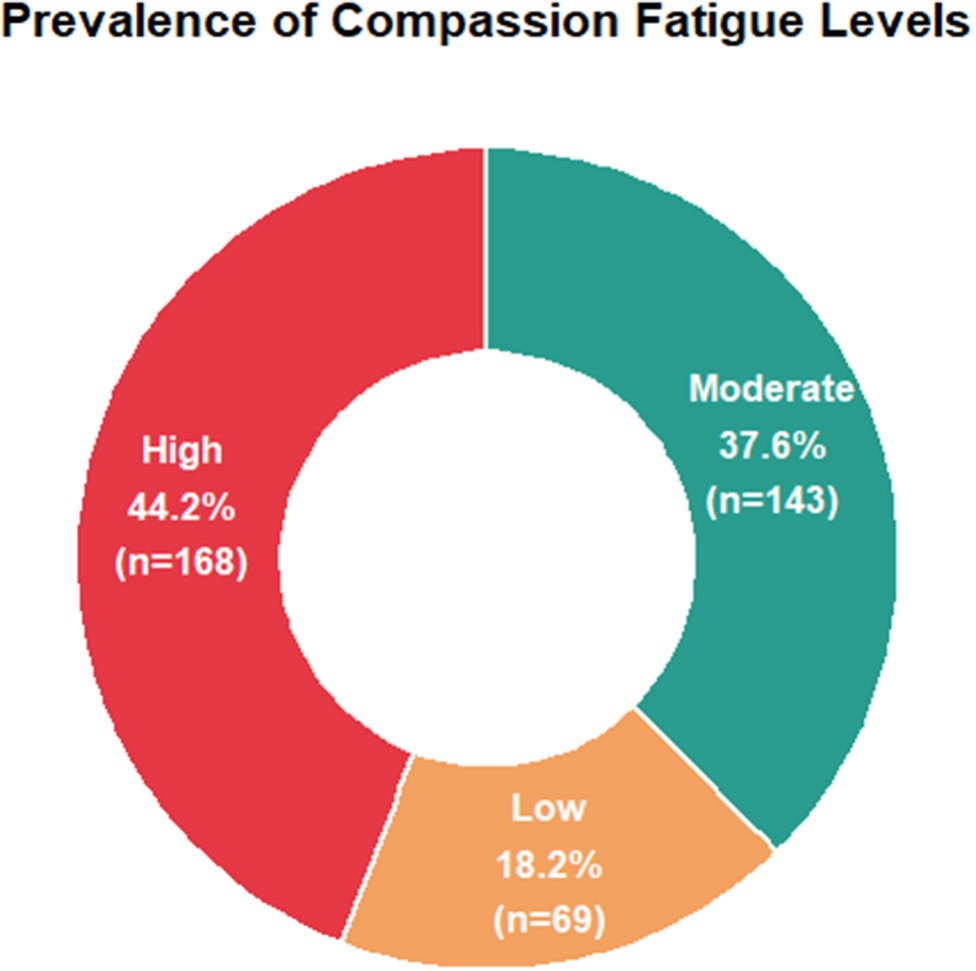
Prevalence of compassion fatigue levels

The overall distribution of compassion fatigue levels among the participants is presented in Fig. 3, highlighting the substantial proportion of nurses affected by high compassion fatigue.

### Departmental variation in compassion fatigue

The prevalence of compassion fatigue varied across clinical departments. Nurses working in Intensive Care/Emergency units exhibited the highest prevalence of high compassion fatigue (52%). This was followed by pediatrics (45%), surgery (40%), and medicine (38%).

**Fig. 4:**
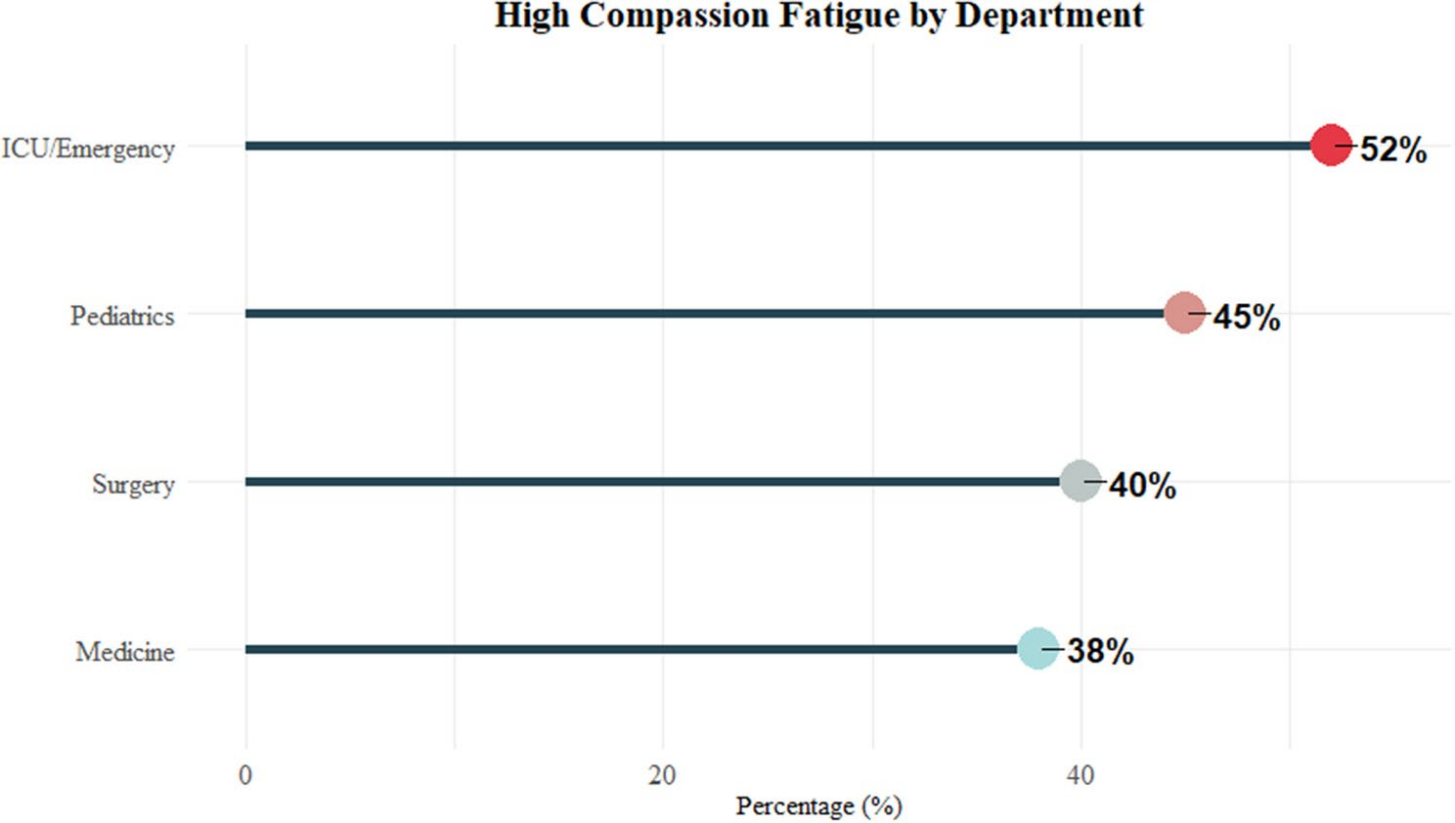
High compassion fatigue by department

**Fig. 5:**
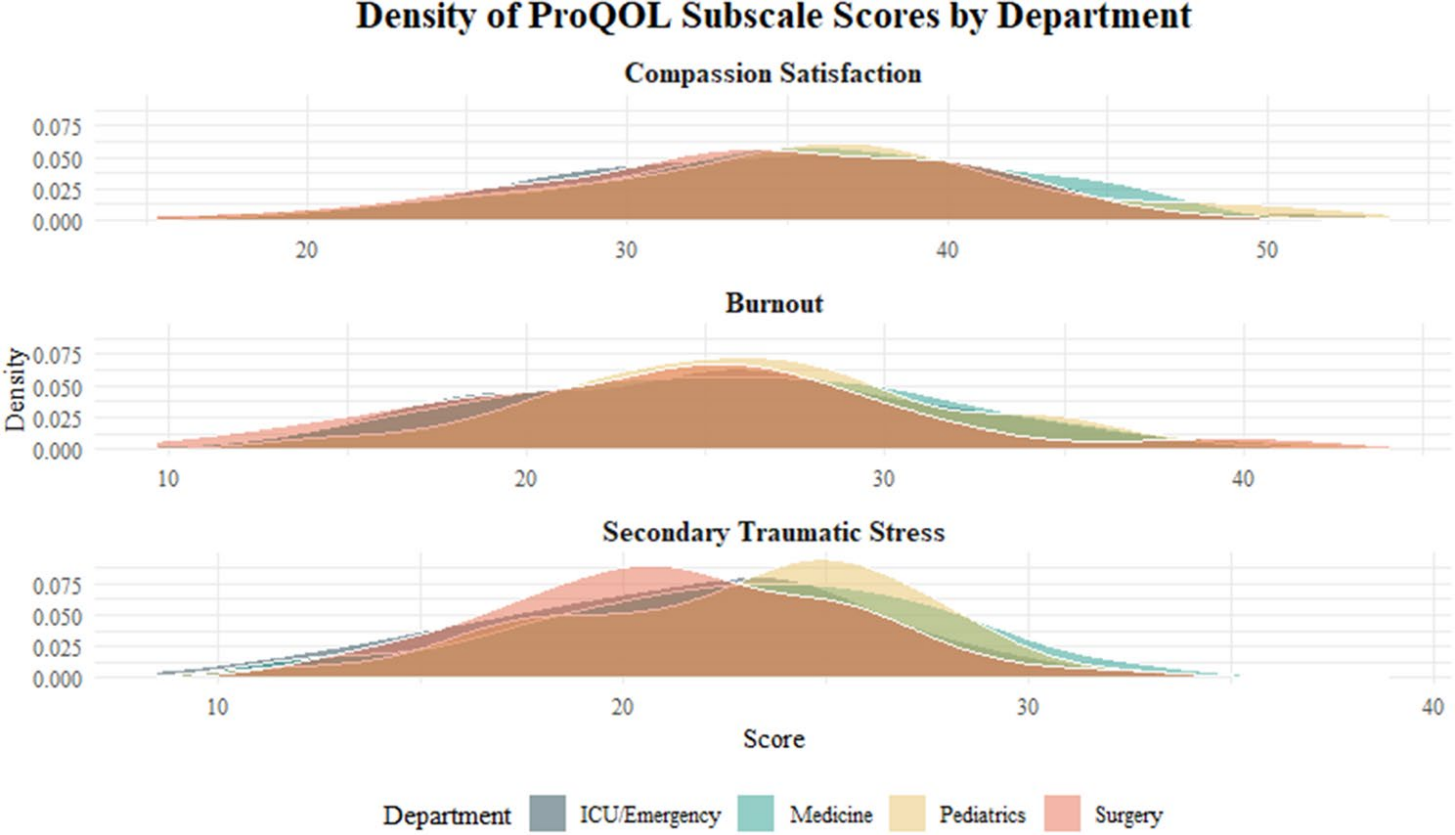
Density of ProQOL subscale scores by department

The distribution of high compassion fatigue across departments is illustrated in Fig. 4. The density distributions of ProQOL subscale scores by department (Fig. 5) further illustrate that nurses in the Intensive Care/Emergency and Surgery departments experienced lower compassion satisfaction alongside higher burnout and secondary traumatic stress than nurses in the Medicine and Pediatrics departments.

### Risk factors associated with high compassion fatigue

Nurses reporting lack of organizational support had 2.6 times higher odds of experiencing high compassion fatigue, indicating a strong positive association between inadequate workplace support and emotional strain. Similarly, nurses with more than 10 years of service demonstrated increased likelihood of compassion fatigue (OR 2.3), suggesting a cumulative exposure effect. Assignment to intensive care/emergency units was also associated with elevated risk (OR 1.9), reflecting the higher emotional demands of high-acuity settings.

Lack of organizational support emerged as the strongest predictor of high compassion fatigue (OR 2.6, 95% CI 1.6–4.1, *p* < 0.001). These findings are summarized in Table 2, summarizes the prevalence and predictors of compassion fatigue among nurses. The confidence intervals did not cross unity, indicating statistically significant associations.

The multivariate model was adjusted for key demographic and occupational variables, including age, sex, department, and years of service.

**Table 2:** Risk factors associated with high compassion fatigue

Factor	OR (95% CI)	*P*-value
Years of service > 10	2.3 (1.4–3.8)	0.002
ICU/Emergency role	1.9 (1.2–2.9)	0.005
Lack of support	2.6 (1.6–4.1)	< 0.001

**Fig. 6:**
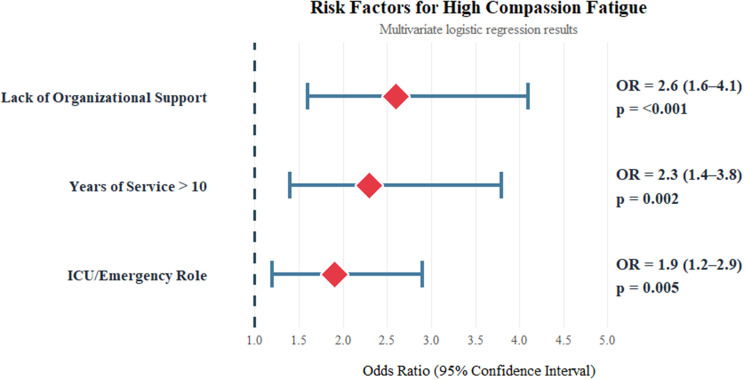
Risk factors for high compassion fatigue

**Fig. 7:**
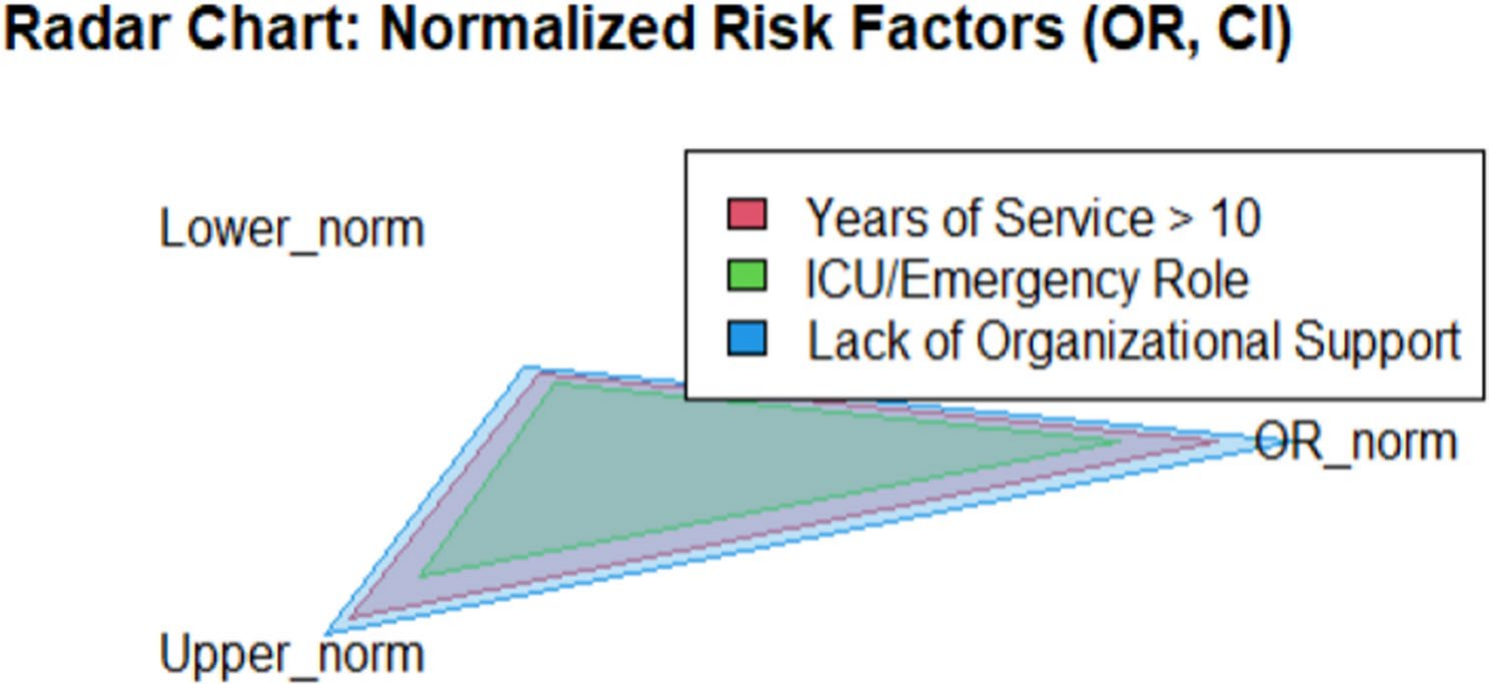
Radar chart of normalized risk factors (OR, CL)

The results are visually represented in the forest plot (Fig. 6) and radar chart (Fig. 7).

### Item-level ProQOL analysis

An item-level analysis of the selected ProQOL-5 items provided additional insights into the nurses’ experiences. Responses revealed substantial variability across the items measuring burnout, secondary traumatic stress, and compassion satisfaction. While a proportion of nurses reported strong agreement with compassion satisfaction items, indicating preserved professional fulfillment, there was also notable agreement with burnout and secondary traumatic stress items.

**Fig. 8:**
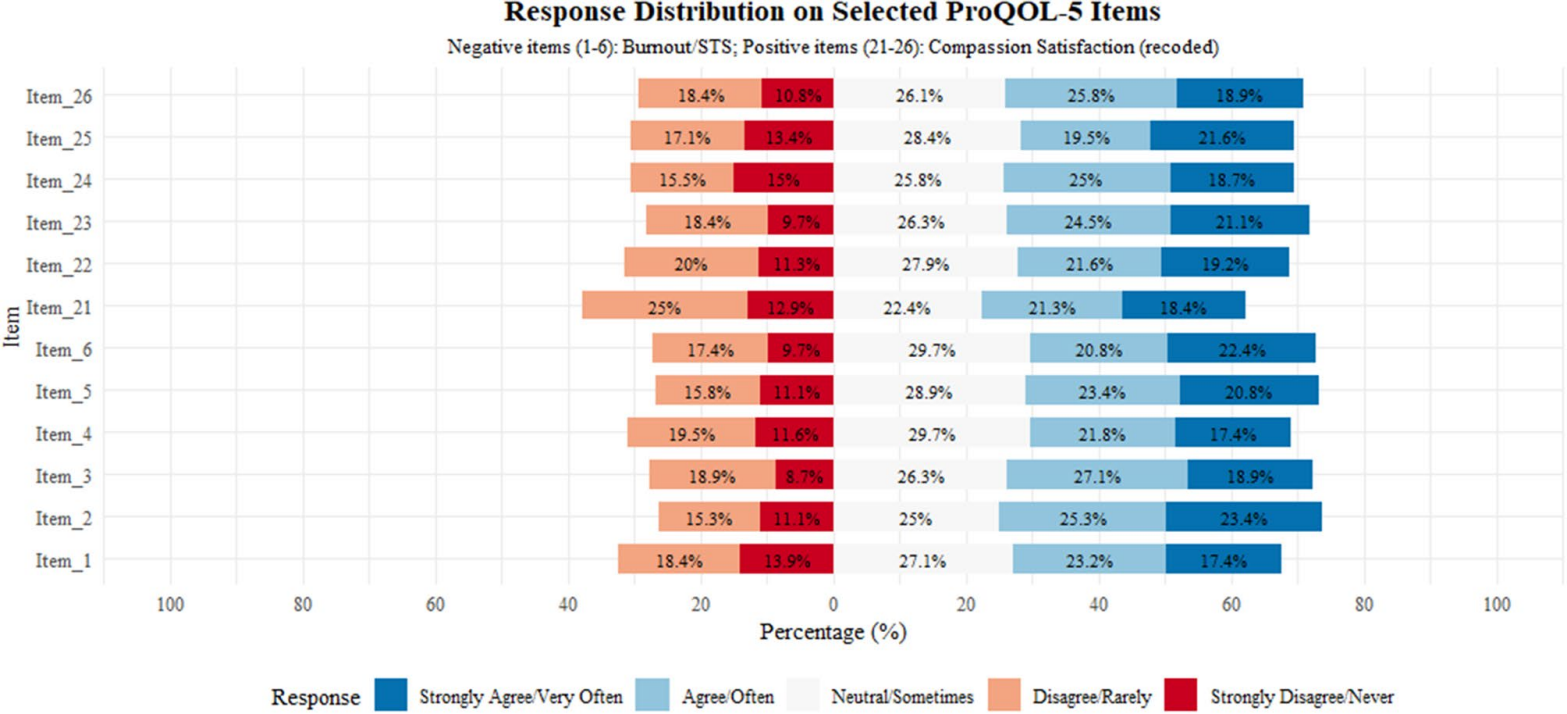
Response distribution on selected ProQOL-5 items

Neutral responses were common across multiple items, suggesting heterogeneous participant experiences. The distribution of responses across the selected ProQOL-5 items is illustrated in Fig. 8, highlighting the coexistence of the positive and negative dimensions of professional quality of life.

## Discussion

This study examined the prevalence and determinants of compassion fatigue among nurses working in a resource-limited district hospital in Bangladesh. The findings demonstrate that compassion fatigue is highly prevalent, with 44.2% of the nurses reporting high levels. This highlights compassion fatigue as a significant occupational concern within district-level public healthcare settings and underscores the emotional burden experienced by nurses who provide continuous care under constrained conditions.

### Interpretation of findings

The prevalence of high compassion fatigue observed in this study is consistent with global reports indicating that approximately 30–50% of nurses experience moderate to high levels of compassion fatigue [4–18]. However, the magnitude observed in this district hospital context is particularly noteworthy, as district hospitals often have fewer resources and support mechanisms than tertiary or specialized healthcare facilities. This suggests that compassion fatigue is not limited to high-technology or specialized units but is also a substantial issue in general public hospitals that serve large populations.

Consistent with the study’s conceptual framework, organizational factors emerged as central determinants of compassion fatigue. Lack of organizational support was identified as the strongest independent predictor of high compassion fatigue. This finding reinforces the view that compassion fatigue is strongly shaped by workplace conditions rather than being solely an individual-level problem, consistent with conceptual models linking organizational factors to healthcare worker burnout and emotional strain [14]. In environments where nurses perceive inadequate managerial support, limited recognition, or insufficient institutional resources, the emotional demands of caregiving may intensify, increasing their vulnerability to compassion fatigue.

Departmental workload intensity was another important factor associated with CF. Nurses working in intensive care and emergency units reported the highest prevalence of compassion fatigue. These findings are consistent with the existing literature, which shows that nurses in high-acuity settings face greater emotional and psychological demands due to frequent exposure to critically ill patients, traumatic events, and time-sensitive clinical decision-making [6, 9]. The elevated burnout and secondary traumatic stress scores observed among nurses in these departments further support the role of sustained exposure to high-stress clinical environments in the development of CF.

Individual and occupational characteristics also contribute to nurses’ vulnerability. Nurses with more than 10 years of professional service were significantly more likely to experience high compassion fatigue, suggesting a cumulative effect of prolonged exposure to caregiving-related stressors. The age distribution patterns observed in this study further support this interpretation, as higher levels of compassion fatigue were more common among relatively older nurses. These findings highlight the interaction between the duration of service and the organizational context in shaping the professional quality of life.

Alternative explanations should also be considered. For example, nurses experiencing higher levels of stress may be more likely to perceive organizational support as inadequate, introducing potential perception bias [14]. Additionally, the association with years of service may reflect cumulative occupational exposure rather than a direct effect of tenure alone.

### Mechanisms underlying compassion fatigue

Subscale and item-level analyses provided additional insights into the mechanisms through which compassion fatigue manifests. Higher burnout and secondary traumatic stress scores among nurses in high-acuity departments indicate that compassion fatigue reflects both emotional exhaustion and indirect exposure to trauma. This supports the multidimensional conceptualization of compassion fatigue proposed by the Professional Quality of Life Model [3].

Simultaneously, the presence of compassion satisfaction among some nurses—even in high-pressure settings—suggests that professional fulfillment and emotional strain may coexist. Item-level findings demonstrated that while many nurses reported indicators of burnout and secondary traumatic stress, a proportion also reported strong agreement with compassion-satisfaction items. This highlights the complex and dynamic nature of nurses’ professional experiences and suggests that compassion fatigue does not uniformly decline in well-being.

These findings imply that effective interventions should not solely focus on reducing stressors but also aim to preserve and enhance sources of professional meaning and satisfaction. Strengthening compassion satisfaction may serve as a protective factor against the negative effects of burnout and secondary trauma stress.

These findings are consistent with the Professional Quality of Life Model, which posits that compassion fatigue arises from the interaction between burnout and secondary traumatic stress, moderated by compassion satisfaction. The observed patterns support the role of organizational and occupational factors in shifting this balance [3].

### Comparison with existing literature

These findings are also consistent with recent post-pandemic evidence highlighting increased psychological burden among nurses [5, 6]. The results of this study are broadly consistent with international research documenting high levels of compassion fatigue among nurses, particularly those working in intensive care and emergency settings [4, 6, 9]. Similar associations between compassion fatigue and years of service, departmental assignment, and organizational support have been reported in studies conducted across diverse healthcare systems [2, 7].

However, this study contributes to the literature by focusing on a district-level public hospital in Bangladesh, a setting that has been under-represented in previous research. Most existing studies in Bangladesh have concentrated on burnout or general mental health outcomes, often within tertiary care or pandemic-related contexts [10–12]. By specifically examining compassion fatigue and its organizational determinants in a district hospital, this study provides context-specific evidence that is directly relevant to the delivery of routine public healthcare.

### Implications for nursing practice and management

These findings have important implications for nursing practice, management, and policy. Given the strong association between organizational support and compassion fatigue, interventions should prioritize system-level strategies rather than relying solely on individual coping mechanisms. Enhancing managerial support, fostering a supportive work environment, and ensuring access to mental health and psychosocial services may help mitigate compassion fatigue among nurses.

Department-specific interventions may be particularly beneficial in high-acuity units, such as intensive care and emergency departments. Strategies such as adequate staffing, workload redistribution, structured debriefing sessions, and peer support programs can help reduce emotional strain in these settings. At the individual level, training programs focusing on resilience, stress management, and self-care may further support nurses’ well-being when integrated within supportive organizational frameworks.

In the context of Bangladesh, these findings highlight the need for strengthening institutional support systems within district hospitals, where workforce constraints and service demand are particularly pronounced.

### Strengths and limitations

A major strength of this study is its relatively large sample size (*n* = 380) and high response rate, which enhance the reliability of the findings. The use of multivariate logistic regression, ProQOL subscale analysis, and item-level exploration adds to the analytical depth and enhances interpretability. Additionally, focusing on district-level public hospitals provides valuable insights into compassion fatigue in resource-limited healthcare settings.

This study has several limitations. The cross-sectional design limits the ability to establish causal relationships between identified predictors and compassion fatigue. The study was conducted in a single district hospital, which may limit the generalizability of the findings to other healthcare settings. Additionally, reliance on self-reported data may introduce response bias. The study also did not account for potential confounding variables such as individual coping strategies, social support, and personality traits, which may influence susceptibility to compassion fatigue. These limitations should be considered when interpreting the findings.

## Conclusion

This study provides empirical evidence of the prevalence and determinants of compassion fatigue among nurses working in a resource-limited district hospital in Bangladesh. The findings indicate that compassion fatigue is highly prevalent, with nearly half of the nursing workforce experiencing high levels of it. This underscores compassion fatigue as a significant occupational concern within district-level public healthcare settings, where nurses routinely manage high patient volumes and complex care demands under constrained resource conditions.

Consistent with the study’s conceptual framework, organizational factors emerged as critical determinants of CF. A perceived lack of organizational support was the strongest predictor, highlighting the influential role of the workplace environment and institutional structures in shaping nurses’ professional quality of life. In addition, nurses working in high-acuity settings, particularly intensive care and emergency units, and those with longer durations of professional service were at an increased risk, suggesting that both cumulative exposure and clinical intensity contribute to their vulnerability.

The findings further demonstrate that compassion fatigue is a multidimensional phenomenon involving burnout, secondary traumatic stress, and compassion satisfaction. The coexistence of emotional strain and professional fulfillment observed in this study indicates that compassion fatigue does not represent a uniform decline in well-being but rather a complex balance between the rewarding and demanding aspects of nursing practice. Addressing compassion fatigue requires approaches that not only reduce stressors but also strengthen sources of professional meaning.

From a practical perspective, the results emphasize the need for organizational-level interventions within district hospitals. Strengthening managerial and peer support, improving workload management, and ensuring access to mental health and psychosocial resources are essential strategies for mitigating CF. Department-specific interventions may be particularly beneficial in high-acuity settings. At the individual level, structured programs aimed at enhancing coping skills, resilience, and self-care may further support nurses’ well-being when embedded within supportive organizational frameworks.

Although limited by its cross-sectional design and single-site setting, this study provides context-specific evidence from a district hospital in Bangladesh, a setting that has been underrepresented in compassion fatigue research. These findings provide a foundation for future multicenter and longitudinal studies to examine causal pathways and evaluate the effectiveness of targeted interventions.

## Data Availability

The datasets generated and/or analyzed during the current study are available from the corresponding author upon reasonable request and are subject to ethical approval.

## Abbreviations

CF: Compassion fatigue
CI: Confidence Interval
ICU: Intensive Care Unit
OR: Odds Ratio
ProQOL: Professional Quality of Life
SPSS: Statistical Package for the Social Sciences
